# Lightweight Power-Line Visual Detection in Agricultural UAV Scenarios Based on an Improved YOLOv12n Model

**DOI:** 10.3390/s26010109

**Published:** 2025-12-23

**Authors:** Yi-Tong Ge, Bao-Ju Wang, Shuai Sun, Yu-Bin Lan

**Affiliations:** Academy of Ecological Unmanned Farm, College of Agricultural Engineering and Food Science, Shandong University of Technology, Zibo 255049, China; 23503030350@stumail.sdut.edu.cn (Y.-T.G.); wbj@sdut.edu.cn (B.-J.W.); 24503030335@stumail.sdut.edu.cn (S.S.)

**Keywords:** object detection, deep learning, power line, agricultural drones, YOLOv12n, dynamic snake convolution

## Abstract

To address the problems of low detection accuracy, slow inference speed, and high computational cost in power-line detection during autonomous operations of agricultural UAVs, this study proposes an improved object detection model based on YOLOv12n. A power-line dataset was constructed using real-field images supplemented with the TTPLA dataset. The lightweight EfficientNetV2 was introduced as the backbone network to replace the original backbone. In the neck, dynamic snake convolution and a multi-scale cross-axis attention mechanism were incorporated, while the region attention partitioning and residual efficient layer aggregation network from the baseline model were retained. In the head, a Mixture of Experts (MoE) layer from ParameterNet was integrated. The improved model achieved 80.07%, 43.07%, and 77.35% of the original model’s parameters, computation, and weight size, respectively. With an IoU threshold greater than 0.5, the mean average precision (mAP0.5) reached 75.5%, representing improvements of 13.53%, 15.09%, 7.5% and 7.54% over YOLOv8n, YOLOv11n, YOLOv5n, and Line-YOLO, respectively. Only inferior to RF-DETR-Nano. On mobile-end testing, the inference speed reached 88.36 FPS and exhibits the highest inference speed across all experimental models. The improved model demonstrates excellent generalization, robustness, detection accuracy, target localization, and processing speed, making it highly suitable for power-line detection in agricultural UAV applications and providing technical support for future autonomous and intelligent agricultural operations.

## 1. Introduction

Agricultural aviation offers advantages such as high operational efficiency, excellent work quality, wide adaptability, low cost, and strong disaster response capabilities [1]. With the rapid development and widespread adoption of agricultural UAVs, autonomous and intelligent spraying operations have become increasingly prevalent. However, the complex and diverse terrain of agricultural environments poses significant challenges to the safe and efficient operation of such drones. Power lines are among the most common obstacles encountered by plant protection UAVs. Due to their low reflectivity, tubular topology, variable scale, and restricted detection cost, UAVs often struggle to accurately detect power lines during flight. This leads to a higher risk of collision, resulting in economic loss and operational hazards. As the demand for autonomous and intelligent navigation continues to rise, achieving accurate and real-time power-line detection on mobile platforms remains a pressing research issue. In recent years, various power-line detection methods based on different sensors have been explored [2]. Millimeter-wave radar was used to acquire obstacle data and combined with a serpent–egret optimization algorithm for classification. 3D LiDAR scanning was applied to detect power lines across mountainous and flat terrains, leveraging point cloud filtering and fitting for extraction. Although radar- and LiDAR-based methods can detect power lines, the large volume of 3D point cloud data makes them impractical for real-time low-altitude UAV operations [3]. A multi-scale LSD algorithm and Markov random field modeling were utilized to identify power-line regions in UAV imagery. Traditional image-processing approaches such as Canny edge detection, Hough transforms, and line-segment detection often struggle with background noise, low visual quality, and high cost, limiting their use in agricultural scenarios. With the rapid advancement of deep learning, neural network-based approaches can now accurately isolate power lines from complex agricultural backgrounds. Lightweight CNN models further enable real-time deployment on mobile platforms [4]. An unsupervised domain adaptation method for transmission line segmentation was proposed, significantly improving detection efficiency [5]. A lightweight network with an FPN structure and Hough transform was introduced to improve line detection under multi-scale conditions [6]. Transformer and DeepLabv3+ were integrated for semantic segmentation, enhancing accuracy and efficiency [7]. LS-NetV2 was proposed, which, based on LSNet [8], was designed to enhance the network’s global feature extraction capability and improve power-line segmentation performance under complex backgrounds by incorporating bipartite matching loss, regression loss updates, and an expanded receptive field [9]. Developed Transformer-based end-to-end detection frameworks achieved state-of-the-art results on benchmark datasets [10]. Existing studies show that, under similar accuracy levels, vision-based sensors outperform radar and LiDAR in terms of computational efficiency and cost [11].

However, most existing research focuses on urban or general environments, leaving agricultural scenarios relatively underexplored. The structural regularity of urban environments, characterized by well-defined object contours and relatively uniform illumination due to reflections from building facades, results in stable and standardized power-line appearances. In contrast, agricultural scenes typically exhibit low-texture backgrounds with high-variance, stochastic features, accompanied by numerous linear noise patterns such as branches and gaps between crops. The openness of these environments further intensifies illumination variability, while the morphology of power lines becomes significantly more diverse. Xie et al. proposed a lightweight power-line detection method designed for low-altitude and complex terrain scenarios, leveraging sub-pixel attention and domain attention mechanisms [12]. Yan et al. introduced a lightweight power-line segmentation network, BA-NetV2, which reduces computational complexity by decreasing the number of parallel branches in the first-generation architecture, while simultaneously enhancing feature extraction capability by expanding the base channel width and incorporating dilated convolutions [13]. The LUM-Net architecture adopts the lightweight EfficientNet-V1 as its backbone and designs a Bi-Large Kernel Convolutional Block (BiLKB) decoder to further improve model efficiency. By integrating a Coordinated Convolutional Block Attention Module, the network strengthens spatial and channel-wise feature focusing, thereby improving detection accuracy [14]. Within agricultural visual perception research, the YOLO family of models has gained widespread use due to its high compression ratio, robustness, and hardware-friendly design. To improve real-time performance on mobile-end UAV platforms, several lightweight adaptations have been proposed. GhostNet modules were employed in YOLOv5s, achieving efficient operation on mobile devices [15]. Inverted residual blocks were integrated and C2f modules were optimized in YOLOv8n to reduce hardware demands [16]. GA-YOLO was designed, in which CBM/CBL were replaced with GBM and SE-CSP structures to improve accuracy and speed [17]. YOLO-PowerLite was proposed, in which C2f_AK and Coordinate Attention mechanisms were introduced, greatly reducing parameters and enhancing adaptability across scales [18]. Power lines, as small and elongated obstacles, occupy only a minimal pixel proportion in UAV imagery [19]. Their high-dimensional features degrade rapidly during deep convolutional processing, making key semantic regions difficult to extract. Additionally, the dynamic lighting, altitude, and viewing angle variations inherent to agricultural environments pose challenges for segmentation robustness. In contrast, object detection offers more flexible adaptability. Under mobile hardware constraints, an efficient power-line detection approach is vital for real-time UAV obstacle avoidance, forming the perceptual foundation for autonomous flight. Given the limitations of current open-source algorithms in balancing speed and accuracy under complex agricultural conditions, this study proposes a novel lightweight detection model, YOLO-PL, based on an improved YOLOv12n architecture [20]. The improvements include: Replacing the backbone with EfficientNetV2 [21]; Enhancing the neck with Dynamic Snake Convolution (DSConv) [22] and Multi-Scale Cross-Axis Attention (MSCAAttention) [23]; Mixture of Experts (MoE) layer in the head from ParameterNet [24]. These enhancements reduce computational cost and hardware dependence while significantly improving detection accuracy and robustness. The proposed model enables real-time and precise recognition of power lines in UAV operational paths, offering reliable perception support for autonomous agricultural UAV navigation.

## 2. Materials and Methods

### 2.1. Power-Line Image Acquisition in Agricultural Environments

**Figure 1:**
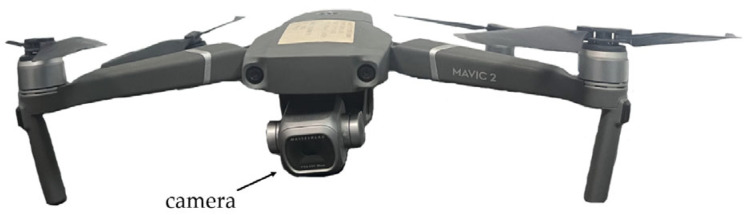
Unmanned Aerial Vehicle (UAV) and camera.

**Figure 2:**
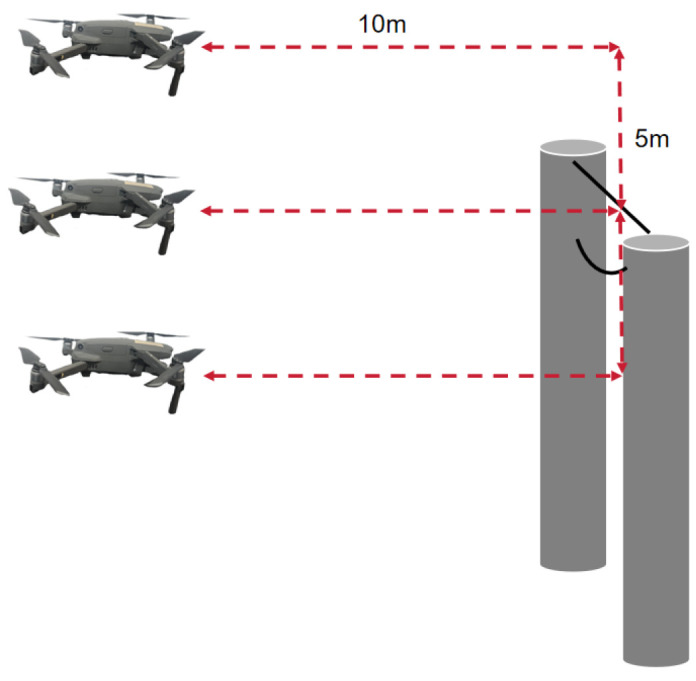
Viewing angles and slant range from UAV to Target.

To obtain representative image data for training and evaluation, field experiments were conducted at Hefeng Farm and surrounding agricultural areas. A UAV was manually controlled to capture RGB images under diverse backgrounds, occlusions, and natural illumination conditions. During data collection, the UAV maintained a horizontal distance of 10 m from the power lines and a vertical distance of 5 m while ensuring both horizontal and vertical camera orientations relative to the lines. The imaging equipment was a DJI Mavic 2 Pro (DJI, Shenzhen, China), with an original image resolution of 5472 × 3648 pixels. The UAV and onboard camera setup are shown in Figure 1, while the imaging geometry and distances are illustrated in Figure 2.

**Figure 3:**
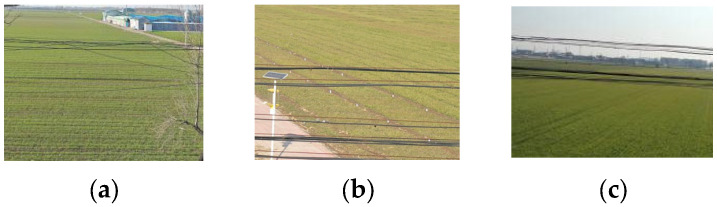
Agricultural environment power-line images. (**a**) Side-lighting with occlusion; (**b**) Back-lighting without occlusion; (**c**) Front-lighting without occlusion.

To improve the model’s robustness to lighting and environmental variations, all images were classified according to occlusion and light source direction (front-lighting, back-lighting, and side-lighting). These categories formed the basis for dataset partitioning. The representative samples are shown in Figure 3, and image classification scheme is summarized in Table 1.

**Figure 4:**
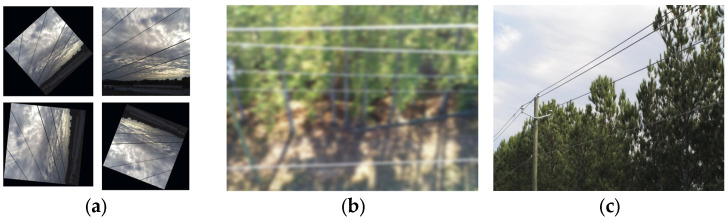
Dataset expansion. (**a**) Image Rotation for Data Augmentation; (**b**) Gaussian Blur; (**c**) TTPLA images.

Because of the high original resolution, direct image compression caused pixel aliasing on the power lines, degrading model training performance. Therefore, cropping was used instead of compression to preserve line continuity. During flight, UAV motion induces variations in the angle and orientation of power lines within the frame. To enhance model robustness to these angular variations, the training set was augmented with rotated images at various angles, improving generalization and reducing overreliance on horizontal patterns. Further augmentations included random scaling, brightness adjustment, and motion blur simulation to mimic UAV sensor motion effects. Additionally, a subset of images from the TTPLA dataset [25] was integrated to improve adaptability to diverse lighting and flight perspectives. After augmentation, the dataset contained a total of 2336 images. Representative augmented samples are shown in Figure 4.

The power transmission lines in the images often appear at various inclination angles. When annotated using LabelImage, the resulting bounding boxes may contain substantial amounts of irrelevant residual information, which undermines the precision of the annotations. To reduce label noise, this study adopts a segmented continuous labeling strategy: based on the morphology and orientation of the power lines, we annotate them using appropriately sized, short sequential segments, allowing for limited and minor overlaps between bounding boxes [26]. In cases where multiple transmission lines are densely adjacent or intertwined within the image, holistic annotation is permitted. The dataset followed the YOLO annotation format. The dataset was divided into training, validation, and test sets in a 6:2:2 ratio, ensuring all lighting and occlusion categories were included in each subset. Training set: 1402 images; Validation set: 467 images; Test set: 467 images.

### 2.2. YOLOv12 Network

The most significant feature of YOLOv12 compared with previous models is the construction of a YOLO framework centered on the attention mechanism, achieving real-time object detection with faster inference speed and higher accuracy. Based on the hierarchical design of YOLOv11, YOLOv12 introduces the Area Attention module (A2) mechanism and the Residual efficient layer aggregation networks (R-ELAN). The overall network architecture of YOLOv12 consists of three main parts: the Backbone, Neck, and Head. The Backbone is primarily composed of convolutional layers, C3K2 modules, and A2C2f modules. The A2C2f module integrates the A2 and R-ELAN structures, which enhance gradient flow and enable faster and more efficient feature extraction. This design is particularly suitable for power lines, which typically exhibit elongated topological structures and spatial distributions spanning across the entire image. In the Neck, the CBS and C3K2 modules are replaced with standard convolutional layers, following the overall architecture of YOLOv12, which improves the efficiency of multi-scale feature fusion for objects of varying sizes. The Head simplifies the multi-scale detection path and introduces a customized loss function designed to better balance localization and classification objectives, thereby improving the real-time performance of the algorithm.

### 2.3. YOLO-PL Network

**Figure 5:**
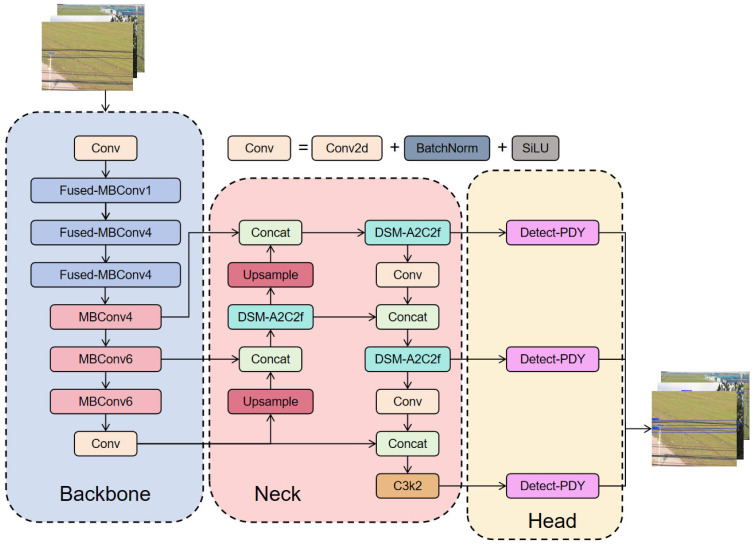
Structure diagram of YOLO-PL.

EfficientNetV2 was introduced as the backbone network, combining MBConv and Fused-MBConv modules and adopting a compound scaling method to enhance network performance, effectively extract image features, and reduce both parameter count and computational complexity. In the neck network, a DSM-A2C2f module was proposed, which integrates DSConv and MSCAAttention while retaining the original A2 and R-ELAN structures. Through a multi-view fusion strategy and a topological continuity constraint loss based on consistent homology, the model adapts to objects at different scales, enhancing its sensitivity to the topological tubular structures of power lines and its multi-scale contextual correlation capability. Finally, an MoE dynamic convolution layer from ParameterNet, referred to as Detect-PDY, was introduced into the head network to further reduce the influence of parameters on floating-point operations. This enhancement improves inference speed and enables a lightweight visual detection model for power lines. The overall network architecture is illustrated in Figure 5.

#### 2.3.1. Backbone Improvement

EfficientNetV2 was introduced to replace the original backbone network of YOLOv12. EfficientNetV2, the second-generation model of the EfficientNet [27] family, retains the core principle of compound scaling, which optimizes the model by jointly adjusting its depth, width, and resolution. Specifically, EfficientNetV2 employs a training-aware neural architecture search (NAS) to determine the optimal parameters for compound scaling. Based on these parameters, the network is proportionally scaled in multiple dimensions to maximize performance while maintaining computational efficiency. This approach enables the model to achieve effective lightweight optimization without sacrificing detection accuracy.

**Figure 6:**
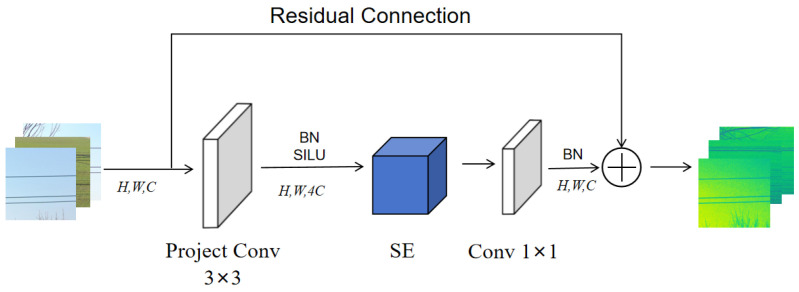
Fused-MBConv module. Note: Project conv is a convolution module with a convolution kernel size of 3 obtained by fusing Conv 1 × 1 and DWConv; *H*, *W*, *C* are the height, width and channel number of the feature map.

**Figure 7:**
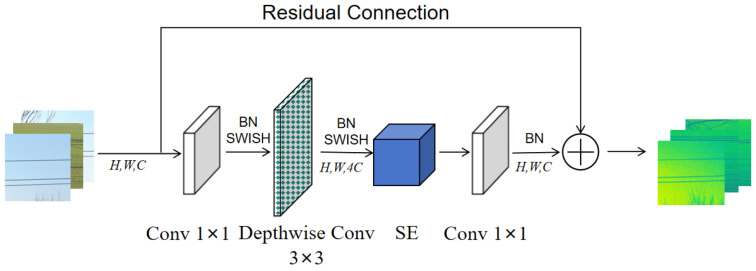
MBConv module.

The MBConv module primarily consists of three components: Expansion Convolution [28], Depthwise Convolution (DWConv) [29], and a Squeeze-and-Excitation (SE) module [30]. Through an inverted residual bottleneck structure, MBConv enhances the nonlinear representation capability of the network, enabling it to extract more expressive features under limited computational resources. However, in the early stages of feature extraction, a large number of depthwise convolutions often fail to fully utilize available computational resources. To address this limitation, the Fused-MBConv module replaces the Expansion Convolution and Depthwise Convolution with a single standard 3 × 3 convolution layer, which achieves further module lightweighting while maintaining the model’s representation power and generalization ability. In the later stages, Depthwise Convolution demonstrates superior performance in high-level feature extraction. Therefore, Fused-MBConv modules are adopted in stages 2, 3, and 4, while MBConv modules are retained in stages 5, 6, and 7 to ensure balanced efficiency and accuracy. The structures of the Fused-MBConv and MBConv modules are illustrated in Figure 6 and Figure 7, respectively.

During the actual improvement process, it was found necessary to prune the channel dimensions of EfficientNetV2. In this work, the automated pruning algorithm MetaPruning [31] is employed to directly generate the pruning strategy. Although the accuracy of EfficientNetV2 decreases by approximately 2–5 points after applying the pruning strategy, its FLOPs are reduced by 60 percent. Compared with the original YOLOv12 backbone, the pruned EfficientNetV2 has a more compact network structure, improving computational efficiency and reducing hardware resource requirements, thereby making it more suitable for deployment on mobile platforms.

#### 2.3.2. DSM-A2C2f Module

This study retains A2 and R-ELAN structures in the neck network. As the core modules of the YOLOv12 architecture, A2C2f integrates both A2 and R-ELAN to enhance the efficiency of feature extraction and aggregation. The Area Attention (A2) module employs a simple yet efficient region-based attention mechanism. Given a feature map of resolution (H, W), it is divided into *i* band-shaped regions of size (H/*i*, W). This operation effectively reduces the receptive field to 1/*i* of the original while still maintaining sufficient coverage for contextual understanding. Within each band region, a multi-head self-attention mechanism inspired by the Transformer [12] architecture is applied. This design reduces the computational complexity of the attention mechanism from O(*n^2^hd*) to O(*n^2^hd*/*i*), thereby significantly accelerating the inference process. At the same time, by dispersing dependence on individual feature channels, it enhances model stability and generalization performance. The computation of the Area Attention mechanism is expressed as Equation (1):

(1) $$\mathrm{Attention(Q,K,V)}=\mathrm{softmax}(\frac{{\mathrm{QK}}^{T}}{\sqrt[{\;}]{d_{k}}})V$$

*Q* is query, *K* is keyword, *V* is value matrix, *d_k_* is keyword dimension, *QK^T^* is the attention map.

ELAN addresses the architectural instability inherent in the Efficient Layer Aggregation Network (ELAN) [32], which often leads to gradient blocking and the absence of residual connections between the input and output layers. To overcome these issues, R-ELAN introduces residual connections spanning from input to output, along with a scaling factor to stabilize feature propagation across layers. In addition, R-ELAN adopts an inverted bottleneck structure, in which transitional channels are first adjusted by an application layer to generate a single feature map, which is then reconnected after passing through subsequent modules. This approach preserves the feature integration capability of the original ELAN while significantly reducing computational cost, parameter count, and memory consumption. Moreover, the use of uniform band-based region partitioning in the Area Attention mechanism avoids the incomplete window-based segmentation problem commonly observed with elongated and horizontally distributed structures such as power lines. This uniform partitioning enables more reasonable attention weight allocation, enhances the efficiency of attention computation, and aligns better with the spatial distribution characteristics of power lines. As a result, the attention mechanism can achieve optimal performance while maintaining low computational complexity.

To address the tubular topological structure and spatial distribution characteristics of power transmission lines, this study introduces DSConv into the original A2C2f module of the neck network. DSConv is specifically designed to be morphologically sensitive to elongated and curvilinear features, allowing the network to better capture the structural continuity of power lines within agricultural imagery. Furthermore, to overcome the limitations of the multi-head self-attention mechanism—namely, sparse attention weights and insensitivity to long-range dependencies—which can cause instability in complex and dynamically changing field environments, this study replaces the traditional multi-head self-attention with MSCAAttention. MSCAAttention enhances cross-directional contextual interactions and strengthens the model’s robustness and adaptability to spatially varying agricultural scenes.

DSConv, proposed in 2023, is a novel convolutional operation designed to enhance adaptability to tubular and topologically continuous structures. By leveraging its adaptive convolutional kernel deformation, DSConv can better conform to the diverse curvilinear and elongated geometries of power lines, thereby improving detection accuracy and avoiding missed or false detections caused by conventional receptive field drift. In the DSConv operation, the feature map is first expanded through a 1 × 1 convolution to increase channel dimensionality. Within the DSConv layer, the standard convolution kernel is dynamically stretched along the *x*-axis and *y*-axis to align with the target’s shape. Taking the *x*-axis as an example, for a given two-dimensional convolution kernel *K*, let the central coordinate be *K_i_* = (*x_i_*, *y_i_*). The position of each grid cell within kernel *K* is defined as follows: each cell corresponds to a horizontal offset *c* = {0,1,2,3,4} from the kernel center. The selection of grid positions in K follows a progressive accumulation process: starting from the central point, the position of each subsequent grid cell is determined by the previous one, incremented by an adaptive offset. This offset is cumulatively added, ensuring that the convolution kernel aligns with the linear or curvilinear structure of the target. The coordinate transformations of Dynamic Snake Convolution along the *x*-axis and *y*-axis are defined by Equations (2) and (3):

(2) $$K_{i\pm c}=\left\{\begin{array}{c} (x_{i+c},y_{i+c})=(x_{i}+c,y_{i}+\sum_{i}^{i+c}\Delta y),\\ (x_{i-c},y_{i-c})=(x_{i}-c,y_{i}+\sum_{i-c}^{i}\Delta y), \end{array}\right.$$

(3) $$K_{j\pm c}=\left\{\begin{array}{c} (x_{j+c},y_{j+c})=(x_{j}+c,y_{j}+\sum_{j}^{j+c}\Delta x,y_{j+c}),\\ (x_{j-c},y_{j-c})=(x_{j}-c,y_{j}+\sum_{j-c}^{j}\Delta x,y_{j-c}), \end{array}\right.$$

**Figure 8:**
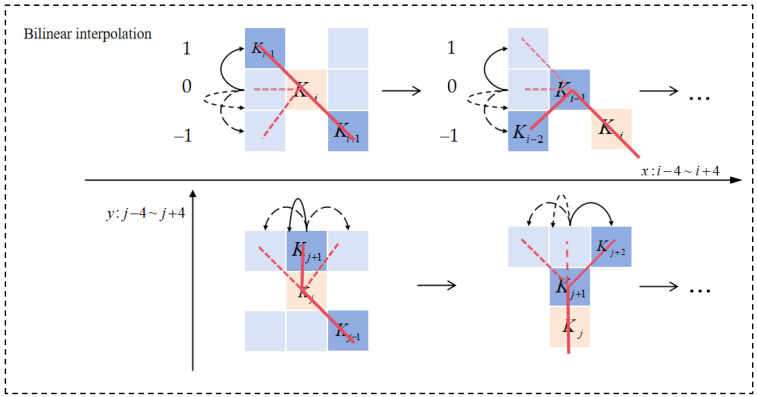
Dynamic Snake Convolution coordinate calculation diagram.

The coordinate computation of the Dynamic Snake Convolution is illustrated in Figure 8:

Since the offsets are typically fractional, bilinear interpolation is used to compute the corresponding feature values, as expressed in Equation (4):

(4) $$K=\sum_{K^{\prime }}B(K^{\prime },K)\cdot K^{\prime }$$

Here, *K* denotes the fractional coordinate positions derived from Equations (3) and (4). *K* enumerates all integer spatial locations within the sampling grid, while *B* represents the bilinear interpolation kernel, which can be decomposed into two one-dimensional kernels, as shown in Equation (5):

(5) $$B\left(K,K^{\prime }\right)=b(K_{x},K_{x}^{\prime })\cdot b\left(K_{y},K_{y}^{\prime }\right)$$

Due to variations along both spatial directions, the Dynamic Snake Convolution covers a 9 × 9 grid region during the deformation process. The adaptive kernel structure of DSConv allows it to better conform to tubular topological geometries, thereby significantly improving the extraction of critical features of power lines.

**Figure 9:**
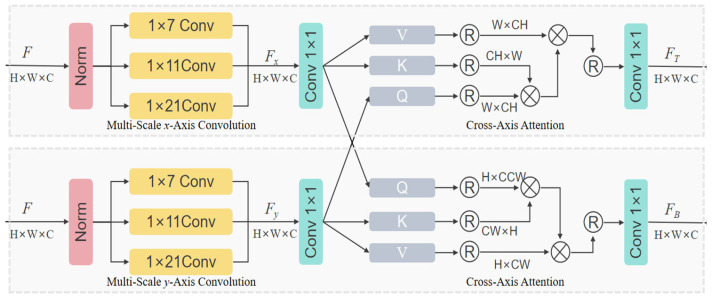
Multi-Scale Cross-Axis Attention module.

The MSCAAttention mechanism computes horizontal and vertical axial attention separately and aggregates features along spatial dimensions through a cross-axis attention mechanism. This enables long-range feature interaction that aligns more closely with the elongated morphology of power transmission lines. The structure of the MSCAAttention module is illustrated in Figure 9.

For a feature map *F*∈*R^H×W×C^*, the MSCAAttention module employs two parallel branches, each consisting of three one-dimensional convolution layers with different kernel sizes to extract multi-scale features. Contextual information is first encoded along one spatial dimension and then aggregated along the orthogonal axis using cross-axis attention. The computation process is expressed in Equation (6):

(6) $$F_{x}=Conv_{1\times 1}(\sum_{i}^{2}Conv_{1\times 1}D_{i}^{x}(Norm\left(F\right)))$$

$Conv_{1\times 1}D_{i}^{x}\left(\cdot \right)$ represents 1D convolution along the *x*-axis, $Norm\left(\cdot \right)$ represents layer normalization, and the other axial *F_y_* can be obtained in the same way.

For the top branch *F_x_*, it is fed into the *y*-axis attention pathway. To more effectively utilize the multi-scale convolutional features extracted along both the *x*-axis and *y*-axis spatial directions for cross-attention computation between *F_x_* and *F_y_*, *F_x_* is designated as the key and value matrices, while *F_y_* serves as the query matrix. The computation process is defined in Equation (7):

(7) $$F_{T}=MHCA_{y}\left(F_{y},F_{x},F_{x}\right)$$

$$F_{out}=Conv_{1\times 1}\left(F_{T}\right)+Conv_{1\times 1}\left(F_{B}\right)+F$$

*MHCA_y_*(·,·,·) refers to the multi head cross attention along the x axis, and the bottom branch *F_B_* is the same.

Compared with the conventional self-attention mechanism, MSCAAttention reduces the computational complexity from O(*HW* × *HW*) to O(*HW* × (*H* + *W*)). Furthermore, by preserving the regional attention partitioning mechanism, its computational load is further reduced to O(*HW*/*i* × (*H*/*I* + *W*)). This enables the module to more effectively capture global contextual information and multi-scale spatial features, minimizing information loss while maintaining a lightweight design. Consequently, MSCAAttention is highly suitable for UAV deployment platforms where computational resources are limited, ensuring both efficiency and accuracy in real-time visual perception tasks.

**Figure 10:**
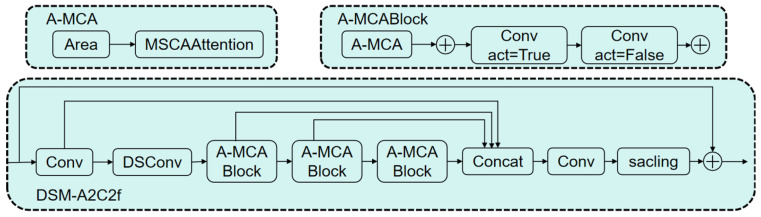
DSM-A2C2f module.

The improved module is designated as the DSM-A2C2f module, and its structure is illustrated in Figure 5. In this module, the input feature map is first passed through a convolution layer for preliminary processing. It then undergoes Dynamic Snake Convolution (DSConv), which adaptively focuses on the local features of tubular structures, thereby enhancing the network’s ability to perceive topological continuity while preserving the morphological characteristics of the target. Subsequently, the feature map passes sequentially through three A-MCA blocks. Each A-MCA block first divides the feature map into iii subregions, then applies a multi-scale cross-axis attention mechanism within each region to strengthen feature representation. The resulting feature maps from all A-MCA blocks are then concatenated and integrated to form a unified representation. Finally, based on the R-ELAN architecture, the original feature map and the integrated feature map are fused, enabling the model to combine global contextual cues with localized structural information, thus improving the overall feature aggregation capability and robustness of the network. The architecture of the DSM-A2C2f module is illustrated in Figure 10.

Through the aforementioned improvements, the neck network is able to more effectively capture and interpret the tubular topological structures and spatial distributions of power lines. These enhancements significantly improve the model’s robustness and detection accuracy for power lines in complex agricultural environments, while also enhancing computational efficiency.

#### 2.3.3. Head Network Improvement

To further lighten the model, a redesigned detection head named Detect-PDY was developed in this study. By integrating dynamic convolution from ParameterNet into the detection head, the computational load on mobile-end platforms is reduced while maintaining detection accuracy. On computation-constrained platforms, rapid inference is typically achieved by significantly reducing the number of parameters to lower floating-point operations (FLOPs). The proposed design enables the model to achieve fast inference and efficient deployment on UAV mobile platforms under a fixed parameter budget and low computational cost.

This dynamic convolution is implemented as a MoE layer, which maintains the same number of parameters while achieving a lower number of FLOPs. The formulation of the dynamic convolution is expressed in Equation (8):

(8) $$Y=X*W^{\prime },W^{\prime }=\sum_{i=1}^{M}=\alpha_{i}W_{i}$$

where $W_{i}\;\in \;R^{Cout\times Cin\times H\times W}$ denotes the *i*-th convolution kernel tensor, and *α_i_* represents the corresponding dynamic coefficient. The coefficient *α_i_* is dynamically generated for each input sample based on the Multi-Layer Perceptron (MLP) module, as shown in Equation (9):

(9) $$\begin{array}{c} \alpha =soft\max(MLP(Pool(X)))\\ \alpha \in R^{M} \end{array}$$

With the increase in parameters, the coefficient generation process in the equation introduces only a minimal increment in FLOPs. By incorporating this dynamic neural network into the convolutional layers of the three detection heads, the overall floating-point operations of the model are significantly reduced, thereby improving inference speed while maintaining detection accuracy.

## 3. Experiments and Evaluation Metrics

### 3.1. Model Training Device and Parameter Setup

Due to the inherent limitations of UAV platforms, the evaluation of the proposed model is divided into training and inference environments to ensure the accuracy and reliability of the experiments. Furthermore, to avoid any dependency on specific mobile hardware vendors, all experiments are conducted without FlashAttention optimization enabled.

The training environment consists of a Windows 11 operating system, 128 GB of RAM, an NVIDIA GeForce RTX 4080 GPU, Python 3.12.8, CUDA 12.6.65, and PyTorch 2.6.0. The inference environment is configured on Ubuntu 20.04, equipped with an NVIDIA Jetson AGX Xavier (32 GB RAM), Python 3.12, and PyTorch 2.4. Based on the dataset scale, the number of training iterations is set to 300, while all other hyperparameters follow the default configuration of the baseline model. The detailed training parameters are summarized in Table 2.

### 3.2. Evaluation Metrics

The evaluation metrics selected in this study include FLOPs, Recall (R), Frames Per Second (FPS), Number of Parameters, and Mean Average Precision (mAP).

FLOPs represent a key indicator of the computational complexity of a model.

It measures the number of floating-point operations required for a single forward propagation, reflecting the computational burden of both training and inference processes.

Recall is used to evaluate the model’s ability to identify all positive samples.

It is defined as the proportion of true positive detections among all actual positive instances, indicating the model’s sensitivity or completeness. where *TP* denotes the number of true positive detections correctly identified by the model, and *FN* denotes the number of false negatives, i.e., positive samples that were not correctly detected.

The formula is expressed as Equation (10):

(10) $$R=\frac{TP}{TP+FN}$$

The inference speed represents the number of image frames processed per second, reflecting the real-time performance of the model. where *T* denotes the total processing time, including preprocessing, inference, and post-processing durations.

It is defined as Equation (11):

(11) $$F=\frac{1}{T}$$

This metric indicates the total number of trainable parameters in the model, which reflects the model’s complexity and storage requirements.

For single-class object detection tasks, the mAP represents the mean precision of the category under different recall levels. mAP0.5 refers to the mean average precision when the Intersection over Union (IoU) threshold is set to 0.5, reflecting model accuracy under relatively relaxed localization conditions. where *N* is the number of classes (in this study, *N* = 1), and AP represents the average precision of class *i* under different recall thresholds. mAP0.5~0.95 denotes the average precision computed across *IoU* thresholds ranging from 0.5 to 0.95, providing a comprehensive evaluation of both detection accuracy and localization performance. The computation is given in Equation (12):

(12) $$mAP_{0.5}=\frac{1}{N}\sum_{i=1}^{N}AP_{i}^{IoU=0.5}$$

## 4. Results

### 4.1. Ablation Experiments

A total of 15 groups of ablation experiments were conducted on the field power-line dataset to evaluate the contribution of each improved component. All ablation experiments were performed on the inference platform, and the experimental results are summarized in Table 3.

As shown in Table 3, in Experiment 2, only the backbone network was replaced with EfficientNetv2, compared with Experiment 1, the model’s FLOPs were reduced by 53.84%, and the inference speed increased by 36.19%. However, mAP0.5 decreased by 6.40%, mAP0.5~0.95 dropped by 7.80%, and R declined by 1.26%. This indicates that the introduction of EfficientNetV2 significantly improves the inference efficiency, but at the cost of a slight reduction in accuracy. The performance degradation is attributed to the channel pruning in EfficientNetV2, which reduces the feature extraction capacity of the network. In Experiments 3, 4, 8, 9, and 14, the incorporation of DSConv led to an overall improvement in mAP0.5. Specifically, in Experiment 8, in Experiment 8, the sole introduction of dynamic snake-shaped convolution raised mAP0.5 and mAP0.5–0.95 by 14.58% and 4.89% relative to the baseline model, respectively, along with improvements in inference speed and recall, while FLOPs decreased. These results demonstrate that DSConv effectively enhances the model’s sensitivity and adaptability to the tubular topological structures of power lines in spatial imagery. In Experiment 7, In Experiment 7, the only integration of the MSCAAttention module primarily improved the mAP0.5~0.95 value, indicating that this attention mechanism enhances the model’s detection performance in complex background environments. In Experiment 5, only MoE of ParameterNet introduced exclusively into the head network reduced the FLOPs from 6.5 G to 5.6 G—a 13.84% decrease—while maintaining nearly identical values of parameters, mAP0.5, mAP0.5~0.95, and recall. In Experiments 6 and 13, when the number of parameters was significantly reduced to approximately 1.9 M, the inference speed increased by 4.64%, and compared with Experiments 1 and 5, the overall inference speed improved by 8.99%. These results indicate that the improved head network effectively reduces computational complexity and enhances inference speed while preserving accuracy and model size. However, as the total number of parameters decreases further, the improvement in inference speed gradually diminishes. In Experiment 15, the improved model achieved a 56.92% reduction in FLOPs, a 41.57% increase in inference speed, a 10.99% gain in mAP0.5, a 16.0% gain in mAP0.5~0.95, and a 0.84% improvement in recall compared with the baseline model. These results demonstrate that the proposed improvements significantly enhance inference efficiency, making the model more suitable for UAV-based deployment, while also improving detection accuracy, localization precision, and robustness in complex agricultural environments. Overall, the ablation results validate the effectiveness of the proposed architectural enhancements. Overall, the ablation results validate the effectiveness of the proposed architectural enhancements.

### 4.2. Model Evaluation Experiment

To verify the performance superiority of the proposed YOLO-PL model in power-line detection for agricultural UAV field operations, as line segment-based detection is inherently dedicated to line segment localization and cannot directly generate power-line instances, this study selects models within the same object detection category. This study selects YOLOv8n, YOLOv11n, YOLOv5n, Line-YOLO [33] and RF-DETR-Nano [34]. Both YOLOv8n and YOLOv5n achieve efficient object detection through streamlined architectures, demonstrating excellent generalization capability across diverse environments and data distributions. They also exhibit strong robustness, maintaining stable detection performance under certain levels of noise and complex environmental conditions, and are widely adopted in various applications. In contrast, YOLOv11n exhibits minimal differences from YOLO-PL in terms of model parameters and computational complexity, allowing us to conduct a more precise and objective comparative analysis of their performance gaps. This allows for a more comprehensive evaluation of YOLO-PL’s real-world effectiveness in detecting power lines under agricultural UAV field conditions. Both Line-YOLO and YOLO-PL belong to YOLO-series models dedicated to power-line detection tasks. RF-DETR-Nano, which is the most lightweight version of RF-DETR. To ensure a more objective and accurate evaluation of the model’s detection performance, no pre-trained weights were used in any of the experiments. The training parameters are listed in Table 2. The model parameter configurations and experimental results are summarized in Table 4.

As shown in Table 4, YOLOv5n possesses the smallest weight file size and the fastest inference speed among YOLOv8n, YOLOv11n, and RT-DETR-R18, reaching 67.23 FPS. Its mAP0.5 and mAP0.5~0.95 remain stable; however, it exhibits the lowest recall rate, which can be attributed to several factors:

1. excessive downsampling, causing feature loss for fine power-line structures;
2. the Anchor-Based mechanism, which shows poor adaptability to elongated targets, limiting its ability to match the shape of power lines;
3. the Focus structure, while reducing computational cost, introduces additional noise, slightly degrading accuracy.

Both YOLOv11n and YOLO-PL have similar parameter sizes, weight file sizes, and FLOPs, and both incorporate attention mechanisms. Compared with YOLOv11n, YOLO-PL outperforms YOLOv11n in overall experimental results. These results confirm the effectiveness of the A2 and R-ELAN modules, demonstrating their superior feature aggregation and attention modeling capabilities. The improved YOLO-PL model achieves the lowest parameter count and FLOPs, with a weight file size of only 4.1 MB, slightly larger than YOLOv5n. It also exhibits the fastest inference speed, exceeding that of YOLOv5n by 31.42%. The model achieves a recall rate of 71.8%, with its mAP0.5 and mAP0.5~0.95 reaching 75.5% and 60.9%, respectively. Compared with Line-YOLO, YOLOv12n-PL outperforms the latter across all metrics on the whole. It is inferred that such a performance gap stems from YOLO-PL’s stronger feature perception capability for irregularly oriented and intertwined power lines in images; meanwhile, this model may not be adaptable to agricultural UAV imagery captured from flying perspectives. RF-DETR-Nano has the largest weight size primarily due to its adoption of the DINOv2 visual encoder. Its mAP0.5 reaches 77.1%, ranking the highest among all tested models. However, its inference speed is excessively slow (only 43.87 FPS), failing to meet the requirements of real-time processing. In addition, its mAP0.5~0.95 is lower than that of YOLO-PL, which suggests that it struggles to suppress linear noise in the background. Overall, the proposed YOLO-PL achieves superior detection accuracy and inference speed while maintaining a compact architecture. Its outstanding performance demonstrates strong robustness and adaptability, providing reliable real-time power-line detection capability for autonomous UAV operations in complex agricultural environments.

**Figure 11:**
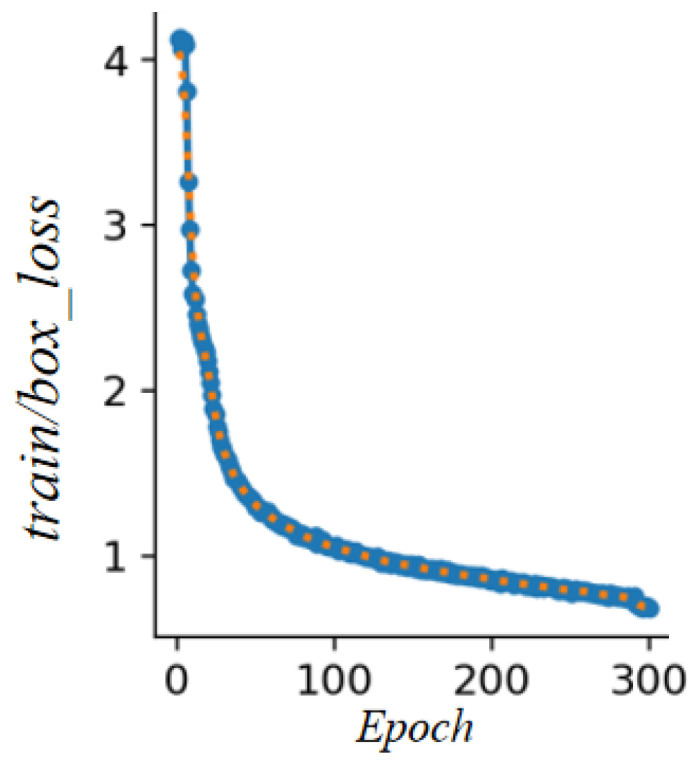
YOLO-PL training box_loss value.

Since the proposed model is primarily designed as a vision-based obstacle avoidance detection system, it requires high localization accuracy for target objects. Therefore, the positional accuracy of power-line targets must be further validated. Considering this requirement, the box loss—a loss function that measures the discrepancy between the predicted bounding box and the ground-truth bounding box—is employed to evaluate the localization capability of the YOLO-PL model during the training and learning process. The results are illustrated in Figure 11.

As shown in Figure 11, the box loss of the YOLO-PL model exhibits a clear downward trend throughout the training process and ultimately stabilizes below 1.0. This indicates that the model’s bounding box regression capability continuously improves and remains stable, demonstrating that the current network architecture and training strategy meet the positional accuracy requirements for power-line detection.

**Figure 12:**
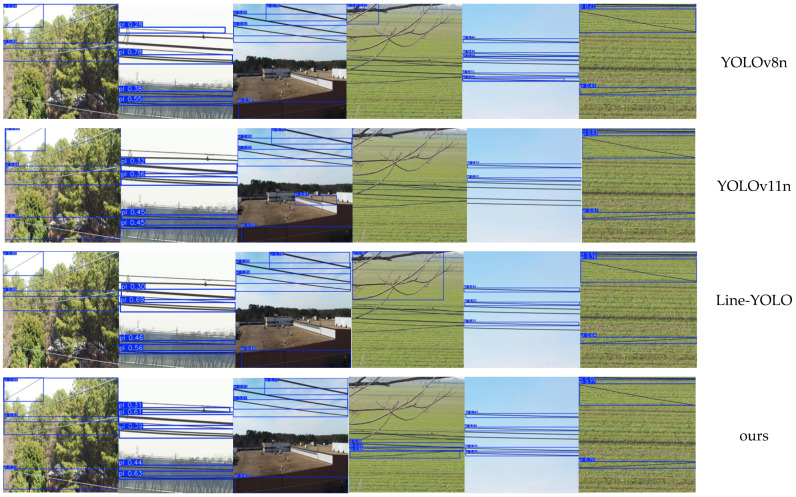
Comparison of experiment results for the model.

Representative detection results of the comparative models are illustrated in Figure 12.

As can be observed, the YOLO-PL model demonstrates excellent robustness and generalization ability in complex and variable agricultural environments. Under challenging conditions such as non-uniform illumination, background interference, partial occlusion, geometric randomness of power-line shapes, and multi-scale target distributions, the model consistently achieves accurate and reliable detections, outperforming other tested models in both accuracy and efficiency. Under varying lighting conditions, YOLO-PL maintains a stable recall rate, effectively suppressing missed and false detections caused by illumination drift. In scenarios with dense crop canopies, gaps, and local occlusions, the model successfully achieves high-confidence separation between the power lines and the background. Regarding the geometric randomness of power-line morphology, YOLO-PL leverages the Dynamic Snake Convolution (DSConv) and Multi-Scale Cross-Axis Attention (MSCAAttention) mechanisms to accurately capture orientation and structural continuity of elongated power lines. Furthermore, during UAV flight, where relative motion causes dynamic scale variations, the model’s multi-scale feature fusion strategy ensures consistent robustness and localization accuracy at different relative distances. Overall, these results confirm that YOLO-PL provides significant performance advantages in power-line detection tasks for agricultural UAVs, offering high-precision and low-latency perception essential for intelligent obstacle avoidance and autonomous flight safety.

**Figure 13:**
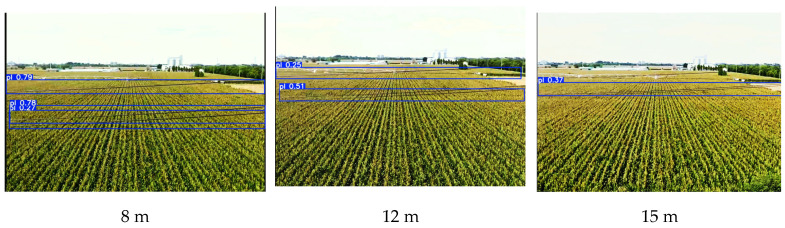
Detection results within the warning zone at maximum operating speed.

To evaluate the impact of UAV flight speed on the detection performance of the proposed model, real flight video streams were collected in actual agricultural field environments. According to relevant aviation regulations, the maximum operational speed of agricultural UAVs must not exceed 50 km/h. Therefore, during data collection, the UAV’s flight speed was maintained at 50 km/h, starting from the boundary of the warning zone [17]. The recorded video sequence has a total duration of 1.2 s, consisting of 68 consecutive frames. To ensure experimental authenticity, the detection tests were conducted entirely on the inference platform, with UAV positions estimated based on frame timestamps. The experimental results show that the model processed the entire video stream in 0.77 s, which is shorter than the video duration, fully meeting the real-time inference requirement. At the maximum flight speed, the model successfully achieved initial detection at the warning zone boundary. When the relative distance decreased by infer 12 m, the detection results became stable, effective, and continuous, and when the UAV approached 8 m, the detection accuracy further improved. In summary, these results demonstrate that the proposed model can provide stable, reliable, and real-time power-line perception within the warning zone, even under the maximum UAV flight speed. Representative detection results are shown in Figure 13.

## 5. Discussion and Conclusions

### 5.1. Advantages

This study aims to develop a power-line detection model for agricultural UAVs that achieves high inference speed, high detection accuracy, and robustness under complex illumination conditions on resource-constrained mobile platforms. Using YOLOv12 as the baseline architecture, the proposed model replaces the backbone with EfficientNetV2 and introduces DSConv, MSCAAttention and dynamic convolution from ParameterNet, while retaining the area attention mechanism and residual inverted structure. Through a series of ablation and comparative experiments, the following conclusions are drawn:

1. The proposed YOLO-PL model enhances detection capability and robustness while improving inference speed and reducing computational cost, by integrating specialized convolutional layers tailored to the geometric characteristics of power lines. Experimental results on the inference platform show that YOLO-PL outperforms the baseline model in terms of inference speed, dataset scalability, recall, and mAP. The model achieves an average detection accuracy of 75.5%, an inference speed of 88.36 FPS, and 2.8 GFLOPs, making it more suitable for deployment and application on UAV-based mobile platforms compared to the baseline model.
2. Compared with other mainstream lightweight models, under identical experimental conditions, YOLO-PL demonstrates significant advantages in lightweight efficiency, detection precision, localization accuracy, and robustness, exhibiting superior generalization ability during testing. The ablation experiments further confirm that each proposed module contributes positively to model performance. Although model lightweighting may slightly reduce detection accuracy, the incorporation of Dynamic Snake Convolution and Multi-Scale Cross-Axis Attention in the neck network compensates for this loss and enhances both detection robustness and localization precision without increasing computational cost. In comparative experiments, YOLO-PL achieves frame rate improvements of 25.74, 21.13, 28.05, 13.91 and 44.49 FPS, mAP0.5 improvements of 9.0%, 9.9%, 11.2% and 5.3%, exhibits a mere 1.6 percentage point reduction relative to RF-DETR-Nano, and mAP0.5~0.95 improvements of 9.3%, 10.5%, 9.6%, 5.2% and 1.6% over other YOLO lightweight models. These results confirm that YOLO-PL satisfies the computational constraints of UAV platforms, achieving faster detection and accurate localization performance in complex and variable agricultural environments and across diverse operational modes, providing reliable visual perception for autonomous power-line avoidance in UAV applications.

### 5.2. Future Perspectives

The proposed model addresses power lines—currently among the most challenging obstacles for UAV visual perception systems—achieving promising results in detection under real-world agricultural conditions. However, compared with more common obstacles such as houses or trees, there remains room for improvement in terms of detection accuracy and generalization capability. Future work will draw inspiration from the Single Feature Enhancement and Temporal Recurrence Network (SETR-Net) [35]. Since agricultural UAV cameras and flight-control systems provide continuous image frames together with positioning and pose information, a streamlined neural-network architecture may be developed to model the temporal relationships among these data sources and enable mutual error correction. This approach has the potential to improve UAV pose and localization accuracy, as well as target detection performance. We will continue to expand the dataset across different environmental conditions and integrate multi-sensor data fusion to further optimize model performance and enhance detection reliability in diverse agricultural scenarios.

## Figures and Tables

**Table 1:** Image classification.

Obscuration	Front-Lighting	Back-Lighting	Side-Lighting
Yes	212	208	459
No	387	301	769

**Table 2:** Training parameters.

Training Parameters	Value
Image Size	1 × 3 × 640 × 640
Number of Iterations	300
Batch Size	32
Initial Learning Rate	0.001
Weight Decay Coefficient	0.0005
Momentum	0.937

**Table 3:** Comparison of YOLO-PL ablation experiment results.

NO.	EfficientNetV2	DSConv	MSCA Attention	ParameterNet	FLOPs (G)	R (%)	FPS (s^−1^)	P (×10^6^ M)	mAP0.5 (%)	mAP0.5~0.95 (%)
1	×	×	×	×	6.5	71.2	62.41	2.56	67.2	52.5
2	√	×	×	×	3.0	70.3	85.00	1.90	62.9	48.4
3	√	√	×	×	2.7	71.2	85.14	1.96	74.7	56.9
4	√	√	√	×	2.7	70.5	85.50	1.96	75.2	58.5
5	×	×	×	√	6.2	70.4	68.02	2.54	67.3	51.4
6	√	×	√	×	3.1	69.7	84.48	1.90	65.1	54.8
7	×	×	√	×	6.5	69.2	62.03	2.22	70.5	55.3
8	×	√	×	×	6.3	71.4	64.22	2.52	77.4	55.2
9	×	√	×	√	4.7	67.0	70.03	2.52	74.8	54.8
10	×	×	√	√	5.5	68.5	65.72	2.24	66.7	53.8
11	√	×	×	√	2.8	67.3	84.82	1.90	60.1	48.1
12	×	√	√	×	6.2	70.6	64.08	2.50	77.7	58.3
13	√	×	√	√	2.8	65.7	88.40	1.91	63.9	45.8
14	×	√	√	√	5.5	66.8	69.00	2.22	73.4	59.7
15	√	√	√	√	2.8	71.8	88.36	1.92	75.5	60.9

**Table 4:** Parameters of experiment models and Comparison experiment results Model.

	Parameter	FLOPs (G)	Model Size (MB)	mAP0.5 (%)	mAP0.5~0.95 (%)	R (%)	FPS
YOLOv8n	3.16	8.9	6.2	66.5	51.6	68.3	62.62
YOLOv5n	4.24	11.9	3.9	65.6	50.4	65.6	67.23
YOLOv11n	2.59	6.4	5.3	64.3	51.3	66.9	60.31
Line-YOLO	5.22	24.3	7.3	70.2	55.7	69.2	74.45
RF-DETR-Nano	5.86	11.3	357.5	77.1	59.3	74.6	43.87
YOLO-PL	2.05	2.8	4.1	75.5	60.9	71.8	88.36

## Data Availability

The raw data supporting the conclusions of this article will be made available by the authors on request.
